# Effect of dietary fiber and threonine content on intestinal barrier function in pigs challenged with either systemic *E. coli* lipopolysaccharide or enteric *Salmonella* Typhimurium

**DOI:** 10.1186/s40104-020-00444-3

**Published:** 2020-04-15

**Authors:** Michael O. Wellington, Kimberley Hamonic, Jack E. C. Krone, John K. Htoo, Andrew G. Van Kessel, Daniel A. Columbus

**Affiliations:** 1 Prairie Swine Centre, Inc., Saskatoon, SK S7H 5N9 Canada; 2 Department of Animal and Poultry Science, University of Saskatchewan, Saskatoon, SK S7N 5A8 Canada; 3 Evonik Nutrition & Care GmbH, Hanau-Wolfgang, Germany

**Keywords:** Barrier function, *E. coli* lipopolysaccharide, Fiber, Goblet cells, Mucin, *Salmonella*, Swine, Threonine

## Abstract

**Background:**

The independent and interactive effects of dietary fiber (DF) and threonine (Thr) were investigated in growing pigs challenged with either systemic *E. coli* lipopolysaccharide (LPS) or enteric *Salmonella* Typhimurium (ST) to characterise their effect on intestinal barrier function.

**Results:**

In experiment 1, intestinal barrier function was assessed via oral lactulose and mannitol (L:M) gavage and fecal mucin analysis in pigs challenged with *E. coli* LPS and fed low fiber (LF) or high fiber (HF) diets with graded dietary Thr. Urinary lactulose recovery and L:M ratio increased (*P* < 0.05) during the LPS inoculation period in LF fed pigs but not in HF fed pigs. Fecal mucin output was increased (*P* < 0.05) in pigs fed HF compared to LF fed pigs. In experiment 2, RT-qPCR, ileal morphology, digesta volatile fatty acid (VFA) content, and fecal mucin output were measured in *Salmonella* Typhimurium challenged pigs, fed LF or HF diets with standard or supplemented dietary Thr. *Salmonella* inoculation increased (*P* < 0.05) fecal mucin output compared to the unchallenged period. Supplemental Thr increased fecal mucin output in the HF-fed pigs (Fib × Thr; *P* < 0.05). Feeding HF increased (*P* < 0.05) VFA concentration in cecum and colon. No effect of either Thr or fiber on expression of gene markers was observed except a tendency (*P* = 0.06) for increased MUC2 expression with the HF diet. Feeding HF increased goblet cell numbers (*P* < 0.05).

**Conclusion:**

Dietary fiber appears to improve barrier function through increased mucin production capacity (i.e., goblet cell numbers, MUC2 gene expression) and secretion (i.e., fecal mucin output). The lack of effect of dietary Thr in *Salmonella*-challenged pigs provides further evidence that mucin secretion in the gut is conserved and, therefore, Thr may be limiting for growth under conditions of increased mucin production.

## Background

A single layer of epithelial cells separates the intestinal lumen from the underlying tissues of the body and plays a key role in responding to changes in the luminal environment [1, 2]. The epithelial layer also functions as a barrier allowing for selective nutrient absorption while preventing toxins, bacteria, and other foreign compounds from entering body circulation. Physical and chemical factors present in the intestinal lumen contribute to the functions of the intestinal barrier [3, 4]. In commercial swine production systems, pigs are exposed to environmental and dietary factors that negatively impact barrier function, affecting their production performance and efficiency [5–7]. Indeed, a compromised intestinal barrier is thought to predispose animals to enteric pathogens and luminal toxins, ultimately inducing inflammation, lowering feed intake and efficiency of feed utilization for growth [8, 9]. Mucus is a glycoprotein secreted by goblet cells that acts to protect the intestinal epithelium from mechanical, chemical and bacterial injuries [10]. Mucus is a major contributor to improved intestinal barrier function, facilitating an unstirred water layer and an environment that limits direct contact of luminal antigens with the epithelium. Dietary fiber (DF) is thought to have both direct and indirect effects on intestinal health and barrier function, including alterations in mucus secretion and cell proliferation as well as changes in the luminal environment as a result of fermentation metabolites [6, 11–13]. However, there are associated negative effects of feeding high DF on endogenous AA losses. Losses of threonine (Thr) are particularly high because mucus contains high amounts of mucin, a Thr rich glycoprotein [14, 15]. Since mucin is largely resistant to digestion, increased secretion of mucus will result in high endogenous losses of Thr [11, 16]. Although mucin secretion is conserved and prioritized [17], there is evidence that mucin secretion might be sensitive to dietary Thr concentration [18]. Previous studies have reported improved intestinal barrier function, increased goblet cell density and increased expression of MUC2 mRNA in pigs and chickens [19–21] when Thr supply was above dietary requirements for growth. Therefore, the objective of this study was to characterize the independent and interactive effects of DF and Thr on markers related to intestinal health and barrier function in pigs during periods of immune stress induced by enteric pathogen challenge (*Salmonella* Typhimurium) or a non-pathogenic systemic model (*E. coli* lipopolysaccharide). It was hypothesized that immune stimulation will negatively affect intestinal barrier function. It was further hypothesized that high DF will improve barrier function due to increased mucus secretion in the gut, regardless of the immune status of growing pigs.

## Materials and methods

### Experimental procedures

#### Experiment 1

As previously reported by Wellington et al. [22], a total of 90 growing barrows (Camborough Plus × C3378: PIC Canada Ltd.) with initial body weight of 20.5 ± 0.75 kg were individually housed in metabolism crates (1.4 m × 1.5 m) in a temperature-controlled room at 20 ± 2 °C at the Prairie Swine Centre, Inc. (Saskatoon, SK, Canada). Briefly, pigs were randomly assigned to 1 of 10 dietary treatments over 9 blocks with a total of 10 pigs per block and 9 pigs per treatment. The dietary treatments were arranged as a 2 × 2 × 5 factorial in a randomized complete block and consisted of a low fiber [LF, 13% total dietary fiber (TDF)] or high fiber (HF, 20% TDF) diet and 1 of 5 levels of dietary Thr [0.49%, 0.57%, 0.65%, 0.73%, and 0.81% standardized ileal digestible (SID)]. The HF diets were formulated by partly replacing corn in the LF diet with 5% wheat bran and 10% sugar beet pulp. Dietary ingredients and inclusion levels represented typical commercial formulations. The experiment lasted for 16 d with an 8-d adaptation period followed by two 4 d collection periods, a pre-ISS period and an ISS period. Immune system stimulation was achieved by intramuscular injection with *E. coli* lipopolysaccharide (LPS; O55:B5, Sigma Aldrich, Oakville, ON, Canada) at an initial dose of 30 μg/kg BW and repeated after 48 h at a 15% increase in dosage [23]. On d 4 of each period, after an overnight fast, pigs were orally dosed using temporary gastric tube (18FR, MED-RX, Canadian Hospital Specialties, Ltd., Oakville, ON, Canada) with a lactulose solution (0.67 g/mL Apo-Lactulose, Apotex Inc. Toronto, ON, Canada) and mannitol solution (0.43 g/mL mannitol, Sigma Aldrich, Oakville, ON, Canada) to provide 0.5 g and 0.3 g/kg BW lactulose and mannitol, respectively. Following the oral gavage, urine was collected over a 24-h period from each pig. The total urine collected was weighed and a 10% aliquot was sampled and stored at − 20 °C for later analysis. Fresh fecal samples were obtained from individual pigs each day during both pre- and post-ISS periods and stored at − 20 °C. At the end of the collection period, fecal samples were pooled and thoroughly mixed for each pig prior to being freeze dried and ground for fecal mucin analysis.

#### Experiment 2

As previously reported by Wellington et al. [24], a total of 128 pigs (Camborough Plus × C3378; PIC Canada Ltd.) of 22.6 ± 1.6 kg initial BW were housed in groups of 4 pigs/pen on solid floors lined with rubber mats in a temperature-controlled room (22 ± 1 °C). Briefly, pens were randomly assigned to 1 of 4 dietary treatments based on the previous study [22] and consisted of an LF (13% TDF) or HF (20% TDF) diet with either a standard (STD; 0.65% SID) or supplemental (SUP; 0.78% SID) Thr level. The HF diets were formulated by partly replacing corn in the LF diet with 5% wheat bran and 10% sugar beet pulp. The experiment lasted for a total of 28 d and consisted of a 7-d adaptation period (unchallenged) and 21 d post-*Salmonella* Typhimurium (ST) inoculation period. On d 0 of the challenge period, pigs were orally inoculated twice within 4 h with a saline solution containing 2.3 × 10^9^ CFU/mL of ST selected to be resistant to the antibiotics Novobiocin (Nov+) and Nalidixic acid (Nal+). Fecal samples were collected from 2 pigs in each pen 2 d before and on d 4 post-ST challenge and stored at − 80 °C and subsequently freeze-dried, ground and mixed thoroughly before subsampling for fecal mucin analysis. On d 7 post-inoculation, one pig/pen representing the average pen BW was humanely euthanized by penetrating captive bolt followed by exsanguination. Subsequently, intestinal tissue (ileum, cecum, and colon) was sampled, snap-frozen in liquid nitrogen, and stored at − 80 °C for RNA isolation and RT-qPCR. Additionally, ileal tissue samples were stored in 10% buffered formalin solution (Thermo-Fisher Scientific Ltd., Toronto, ON, Canada) for tissue morphological analysis. Digesta samples (ileum, cecum, and colon) were collected into 15 mL tubes and stored at − 80 °C for volatile fatty acid (VFA) analysis.

### Analytical procedures

#### *In vivo* barrier permeability analysis (Experiment 1)

Urinary analysis for lactulose and mannitol was completed at the National Research Council (Saskatoon, SK, Canada) using ion chromatography based on the procedure of Hurum and Rohrer [25]. Briefly, the urine samples were diluted (1:100) with deionized water and the mixture inverted several times. An aliquot of 1 mL of the sample mixture was transferred into 1.5 mL polypropylene injection vials. Lactulose and mannitol concentration was analyzed on a Dionex ICS-3000 ion chromatography system (Thermo Scientific, Sunnyvale, CA, USA) using Chromeleon software (version 6.80 SR10, build 2818) with a Dionex CarboPac MA1 4 × 50 mm guard followed by a Dionex CarboPac MA1 BioLC Analytical 4 × 250 mm column (Thermo Scientific, Sunnyvale, CA, USA). The mobile phase was 480 mmol/L NaOH at a flow rate of 0.4 mL/min*.* The detector was programmed to quantify using the calibration curves run with the samples to yield the amount of lactulose or mannitol (μg/mL). Standards were prepared and analyzed with each batch of urine samples.

#### Total fecal mucin analysis (Experiment 1 and 2)

Fecal mucin concentration was analyzed according to methods described previously by Bovee-Oudenhoven et al. [26] with modifications according to the kit manufacturer (Fecal mucin assay kit, #CSR-FFA-MU-K01E, CosmoBio, Ltd., Tokyo, Japan) to quantify N-Acetylgalactoseamine. Fluorescence emission values at 383 nm were recorded following excitation at 336 nm (Synergy™ Multi-Mode Reader, BioTex Instruments, Inc., Vermont, USA), and plotted on a standard curve of N-Acetylgalactoseamine provided in the kit to determine fecal mucin concentration (mg mucin/g of fecal DM). In experiment 1, daily fecal DM output was estimated based on recorded daily feed intake and apparent fecal dry matter digestibility was determined via inclusion of an indigestible marker in the diet [22]. Total fecal mucin output was then calculated via mucin concentration (mg mucin/g fecal DM) corrected for daily fecal DM output. As the same diets were used, the same digestibility value was applied to recorded daily feed intake to estimate total mucin output for Experiment 2.

#### Ileal morphology and goblet cell numbers (Experiment 2)

Ileal tissue samples were obtained 15 cm from the ileocecal junction and immediately fixed in 10% buffered formalin. The fixed intestinal segments were embedded in paraffin and sectioned for intestinal morphology (Prairie Diagnostic Services, Saskatoon, SK). Briefly, sections of the tissue were deparaffinized in xylene, rehydrated and stained with haematoxylin and eosin. Slide images were measured at 10 × magnification using an Axio Star Plus light microscope (Axio Scope A1; Carl Zeiss Gottingen, Germany). The villus height and crypt depth of each tissue were measured using the AxioVision Rel 4.8 software (Carl Zeiss Canada Ltd., Toronto, ON) on a minimum of 10 well-oriented villi and their corresponding crypts per sample. Goblet cell counts were determined by preparing tissue samples as indicated above and staining with Alcian Blue and Periodic Acid Schiff as previously described [27]. The slides were then viewed under a light microscope at 10 × magnification (Axio Scope A1; Carl Zeiss Gottingen, Germany). For each tissue sample, 10 well-oriented villi were selected, and goblet cells were counted in a region within 100 μm length of the villi.

#### Volatile fatty acid analysis (Experiment 2)

Volatile fatty acid analysis followed the procedure by Khorasani et al. [28] and Lenahan et al. [29]. Briefly, digesta samples (ileum, cecum, and colon) were diluted with 25% metaphosphoric acid at a 2:1 ratio (w/v). Samples were centrifuged at 12,000×*g* for 10 min and the supernatant was collected into 2 mL centrifuge tubes and further centrifuged at 16,000×*g* for 10 min. Following that, the supernatant was collected and filtered through 0.45 μm PVDA filter (Fisher Scientific, Hampton, New Hampshire, USA) into 1.5 mL tubes. An internal standard (4.56 μmol/mL isocaproic acid in 0.15 mol/L oxalic acid) was added at 0.2 mL to 1 mL of the filtered sample supernatant and inverted to mix thoroughly. The VFA were determined on an Agilent 6890 gas chromatograph with a flame ionization detector (Agilent Technologies, Santa Clara, California, USA) and a capillary column ZB-FFAP (30 m length × 0.32 mm width × 0.25 μm film thickness; ZEBRON, Phenomenex, Torrance, California, USA). The initial oven temperature was set at 90 °C and a hold time of 0.1 min, then followed by the 1^st^ ramp; 10 °C per minute which increased until 170 °C with a hold time of 6 s. The 2^nd^ ramp was 20 °C per minute up to 230 °C with and a hold time of 2 min. Hydrogen gas was used for the FID and helium gas was used as a carrier.

#### Gene expression analysis (Experiment 2)

Tissue samples stored at − 80 °C were ground in liquid nitrogen with mortar and pestle and total RNA was extracted from tissue samples using TRIzol reagent (Invitrogen, Carlsbad, CA) according to the manufacturer’s protocol. The RNA concentration and quality were determined using a spectrophotometer (NanoDrop 2000 spectrophotometer, Thermo Fisher Scientific Inc., Delaware, USA) with optical density ratio (260:280) between 1.8 and 2.0. The integrity of RNA was then assessed by gel electrophoresis. Reverse transcription was carried out using a high capacity cDNA reverse transcription kit (Applied Biosystems, CA, USA) with random hexamer primers. Each 20 μL of reaction mix contained 0.8 μL of 10 μmol/L primer concentration for each forward and reverse primer, 6.4 μL of nuclease-free water, 10 μL of EVA Green supermix (Bio-Rad Laboratories, CA, USA) and 2 μL (2 ng/qPCR reaction) of template cDNA. Standard curves were made for each gene using a 5-fold serial dilution of pooled cDNA samples from all experimental treatments. PCR efficiency between 90% and 110% were accepted. All genes (Table 1) were analyzed with a standard dilution series prepared from cDNA on the same plate and the starting quantities (recorded as arbitrary values) calculated for each gene on each plate. Arbitrary values for genes of interest were normalized using the mean of the arbitrary values for GAPDH and RPL19, which were not affected by treatment.

**Table 1:** Primers used in quantitative PCR analysis^a^

Gene	Forward (5′ to 3′)	Reverse (5′ to 3′)	AT, °C	NCBI accession number
*RPL19*	AACTCCCGTCAGCAGATCC	AGTACCCTTCCGCTTACCG	60	AF_435591
*MUC2*	ACCCGCACTACGTCACCTTC	GGCAGGACACCTGGTCATTG	62	BX671371
*CLDN4*	CAACTGCGTGGATGATGAGA	CCAGGGGATTGTAGAAGTCG	60	NM_001161637.1
*GAPDH*	CTTCACGACCATGGAGAAGG	CCAAGCAGTTGGTGGTACAG	63	AF017079
*ZO-1*	ACGGCGAAGGTAATTCAGTG	CTTCTCGGTTTGGTGGTCTG	60	XM_003353439.2
*IL8*	TCCTGCTTTCTGCAGCTCTC	GGGTGGAAAGGTGTGGAATG	62	NM_213867
*Casp3*	AGAGGGGACTGCTGTAGAACT	CCGTCTCAATCCCACAGTCC	59	NM_214131.1

^a^*GAPDH* glyceraldehyde 3-phosphate dehydrogenase, *RPL19* ribosomal protein-L19, *ZO1* Zonula Occludin −1,*MUC2* Mucin-2, *CLDN-4* Claudin-4, *IL8* Interleukin-8, *Casp3* Caspase-3, *AT* annealing temperature

### Statistical analyses

Data were tested for normality using the Shapiro-Wilk test (PROC UNIVARIATE, SAS 9.4, SAS Institute Inc., Cary, NC). In experiment 1, all data were analyzed as a 2 × 2 × 5 factorial in a randomized complete block design (PROC MIXED, SAS 9.4, SAS Institute Inc., Cary, NC). The model included main effects of [a] fiber level (High or Low DF), [b] period (ISS or Pre-ISS) [c] threonine level (0.49%, 0.57%, 0.65%, 0.73%, and 0.81% SID) and their interactions and block as a random effect. In experiment 2, VFA, qPCR, and ileal morphology and goblet cell samples were analyzed as 2 × 2 factorial arrangement in a randomized complete block design. The model included the main effects of [a] fiber (High or Low DF) and [b] threonine (STD Thr or SUP Thr) and their interaction and block as a random effect. Data for the fecal mucin output were analyzed as 2 × 2 × 2 factorial arrangement in a randomized complete block design. The model included the main effects of [a] fiber (High or Low DF) and [b] threonine (STD Thr or SUP Thr) and [c] period (pre-ST inoculation and post-ST inoculation) and their interactions and block as a random effect. Significant differences were determined at *P* < 0.05 and a trend toward significance considered at *P* ≤ 0.10. When significance was observed, the means were separated according to the least significance difference (LSD) method (PDIFF option in SAS 9.4, SAS Institute Inc., Cary, NC).

## Results

### *In vivo* barrier permeability (Experiment 1)

**Fig. 1:**
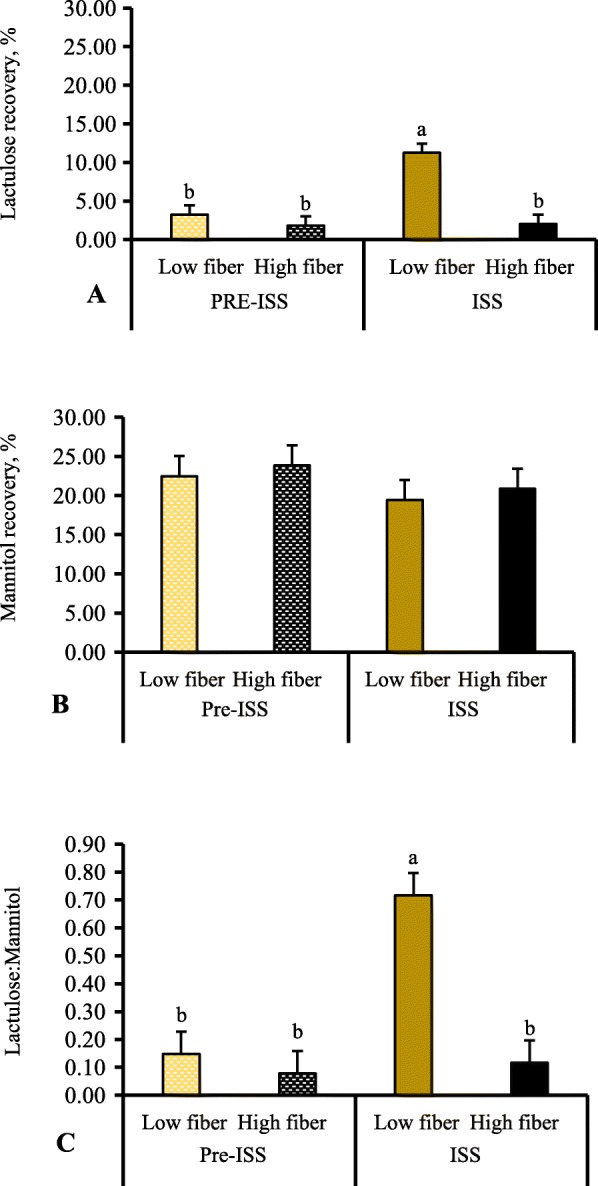
Urinary lactulose (**a**), mannitol (**b**) and lactulose:mannitol ratio (**c**) in *E. coli* lipopolysaccharide challenged and unchallenged pigs fed either high or low fiber diets with graded dietary threonine levels. A total of 9 replicate pigs/treatment were used in the analysis

There was no Thr effect on lactulose or mannitol recovery or lactulose:mannitol **(**L:M) ratio. As shown in Fig. 1, a significant period × fiber interaction (*P* < 0.01) was observed on lactulose recovery and L:M ratio (Fig. 1a and c). When no immune stimulation was present, lactulose recovery and L:M ratio was not different between LF and HF-fed pigs, however following ISS, lactulose recovery and L:M ratio was increased in LF-fed pigs, but not in HF-fed pigs. The recovery of mannitol was not significantly affected (*P* > 0.05, Fig. 1b) by either Thr, fiber or period, with no interaction.

### Total fecal mucin output (Experiment 1 and 2)

**Fig. 2:**
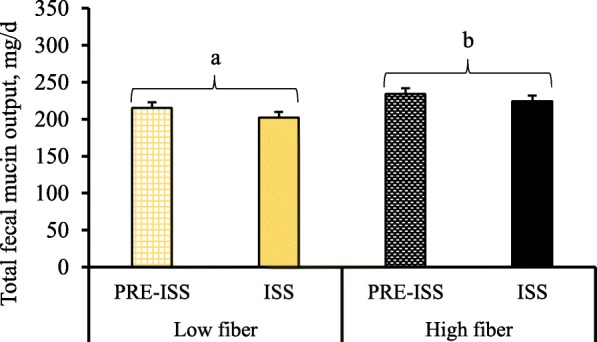
Total fecal mucin output (mg/d) in LPS challenged and unchallenged pigs fed high or low fiber with graded dietary threonine. Total fecal mucin output was estimated using determined mucin concentration in feces and estimated total fecal output based on previously determined dry matter digestibility. A total of 9 replicate pigs/treatment were used in the analysis. There was a significant effect of fiber (*P* < 0.05) with no effect of Thr or period (*P* > 0.05) on total fecal mucin output

In experiment 1, the total fecal mucin output was determined in feces of pigs during pre-ISS and ISS periods and data are presented in Fig. 2. Neither dietary Thr nor ISS by LPS had an effect on total fecal mucin output (*P* > 0.05), however, total fecal mucin output was significantly increased in the HF fed pigs (*P* < 0.05).

**Fig. 3:**
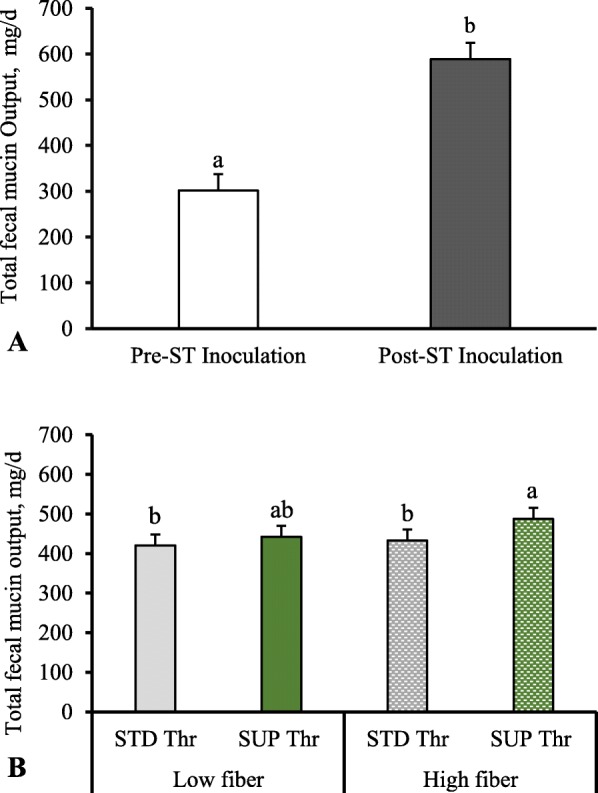
Total fecal mucin output (mg/d) 2 d before and 4 d post-*Salmonella* Typhimurium inoculation in pigs fed high or low fiber diets with either standard or supplemental dietary threonine. Total fecal mucin output was estimated using determined mucin concentration in feces and estimated total fecal output based on previously determined dry matter digestibility. A total of 8 replicate pigs/treatment were used in the analysis. Total fecal mucin output was higher post-ST inoculation (*P* < 0.01) compared to output pre-inoculation (**a**). There was a significant fiber × Thr interaction (*P* < 0.05) on total fecal mucin output (**b**)

In experiment 2, before ST inoculation a lower (*P* < 0.01) total fecal mucin output was observed compared to total fecal mucin output post ST inoculation (Fig. 3a). A fiber × Thr interaction (Fig. 3b; *P* < 0.05) on total fecal mucin output was observed, where Thr supplementation increased total fecal mucin output to a greater extent in pigs fed HF diet compared to LF-fed pigs.

### Ileal morphology and goblet cell numbers (Experiment 2)

**Fig. 4:**
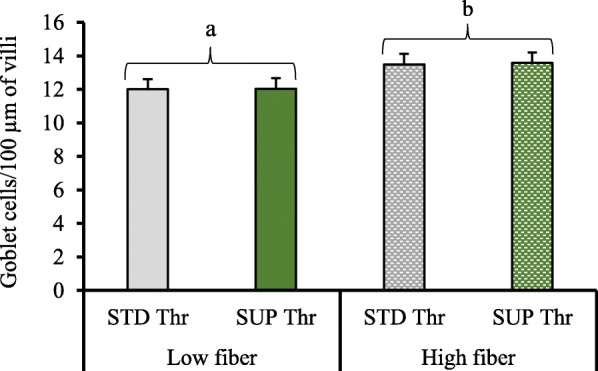
Goblet cell counts (number/100 μm length of villi) of pigs challenged with *Salmonella* Typhimurium. We observed a significant (*P =* 0.04) increase in goblet cell number with high fiber diets. A total of 8 replicate pigs/treatment were used in the analyses

There were no significant effects of fiber, Thr, or an interaction on the villus height, crypt depth, or villus height:crypt depth ratio (*P >* 0.05) in ileal tissue samples of pigs 7 d post-ST inoculation (Table 2). No effect of Thr (*P* > 0.05) was observed on goblet cell numbers, however, goblet cell numbers were increased (*P* < 0.05) in HF-fed pigs compared to the LF-fed pigs (Fig. 4).

**Table 2:** Ileal morphology in pigs’ 7-d post-*Salmonella Typhimurium* inoculation^a^

Item	Low fiber		High fiber		SEM^d^	*P*-value		
	STD^b^ Thr	SUP^c^ Thr	STD Thr	SUP Thr		Fiber	Thr^e^	Fiber × Thr
Villus height, μm	433.9	438.8	446.4	434.2	25.6	0.867	0.859	0.697
Crypt depth, μm	268.7	290.9	313.5	292.7	17.6	0.157	0.963	0.188
VH:CD^f^	1.69	1.59	1.46	1.57	0.10	0.207	0.998	0.271

^a^Total of 8 replicate pigs/treatment (*n* = 8/treatment)

^b^*STD Thr* Standard Thr (0.65% standardized ileal digestible)

^c^*SUP Thr* Supplemental Thr (0.78% standardized ileal digestible)

^d^*SEM* Standard error of the mean

^e^*Thr* threonine

^f^*VH:CD* villus height:crypt depth

### Volatile fatty acid concentration (Experiment 2)

Results for VFA concentration in digesta are presented in Table 3. In the ileal digesta, there were no effects of fiber, Thr or their interaction on the concentration of VFA. In the cecal digesta, HF increased (*P* < 0.05) the concentration of acetate, propionate and the total VFA, but had no significant effect on butyrate concentration (*P* > 0.05). Interestingly, we observed that the total VFA concentration, primarily influenced by the acetate concentration in cecal digesta increased (*P* < 0.01) with SUP Thr. In the colonic digesta, we observed no significant Thr effect (*P* > 0.05) on VFA concentration. However, HF increased (*P* < 0.01) colonic concentration of acetate, with a tendency (*P* = 0.08) to increase butyrate concentration and no effect (*P* > 0.05) on propionate concentration. Total VFA concentration in the colonic digesta was higher (*P* < 0.05) in the HF fed pigs than in LF fed pigs.

**Table 3:** Effect of fiber and threonine on volatile fatty acid concentration in digesta of pigs challenged with *Salmonella* Typhimurium^a^

	Low fiber		High fiber		SEM^d^	*P-value*		
	STD^b^ Thr	SUP^c^ Thr	STD Thr	SUP Thr		Fiber	Thr^e^	Fiber × Thr
Cecum, μmol/g								
Acetate	58.31	66.29	64.41	87.33	4.71	< 0.01	< 0.01	0.089
Propionate	45.02	48.5	55.56	63.86	5.91	< 0.05	0.347	0.699
Butyrate	25.56	29.47	21.47	26.16	2.76	0.248	0.183	0.902
Total VFA^6^	128.9	144.26	141.44	177.35	9.37	< 0.05	< 0.01	0.264
Colon, μmol/g								
Acetate	46.39	45.99	67.69	57.06	7.37	< 0.01	0.319	0.354
Propionate	25.65	25.35	29.01	27.4	4.18	0.473	0.798	0.860
Butyrate	13.34	15.24	18.32	18.42	2.75	0.083	0.659	0.692
Total VFA	85.38	86.65	115.02	102.87	12.93	< 0.05	0.613	0.533
Ileum, μmol/g								
Acetate	13.76	14.2	14.86	12.64	3.66	0.923	0.713	0.583
Propionate	0.1	0.31	0.43	0.54	0.03	0.151	0.381	0.758
Butyrate	0.85	0.7	0.41	0.76	0.03	0.468	0.696	0.328
Total VFA	14.68	15.21	15.69	13.95	4.08	0.961	0.819	0.669

^a^Total of 8 replicate pigs/treatment (*n* = 8/treatment)

^b^*STD* Thr Standard threonine

^c^*SUP* Thr Supplemental threonine

^d^*SEM* Standard error of the mean

^e^*Thr* Threonine

^f^*VFA* Volatile fatty acid

### Gene expression of markers for barrier function and gut health (Experiment 2)

**Fig. 5:**
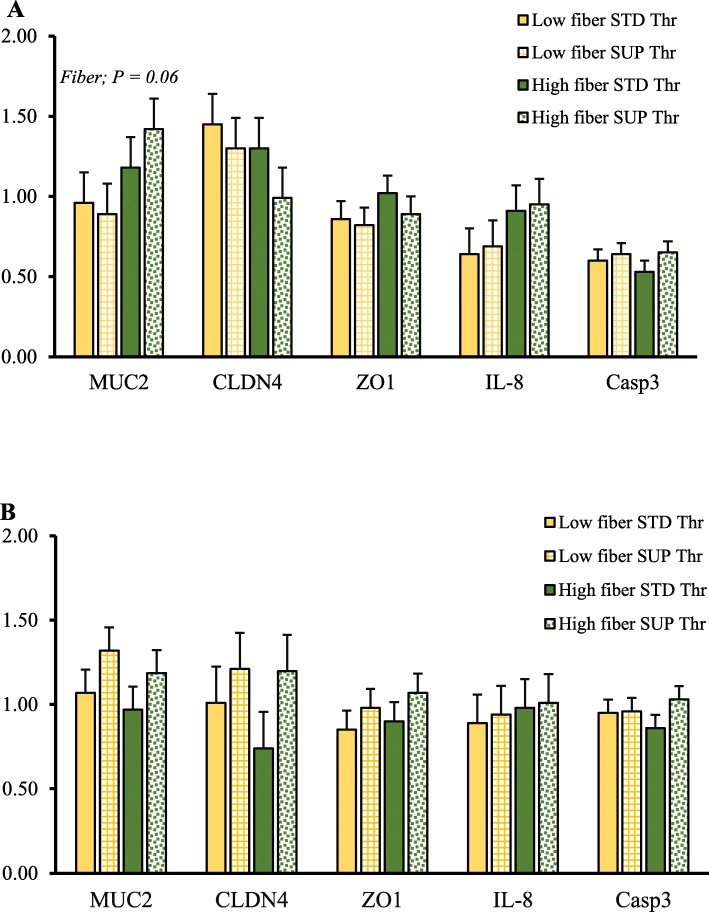
Ileal tissue (**a**) expression of marker genes for intestinal barrier function and colonic tissue (**b**) expression of marker genes for intestinal barrier function and. A total of 8 replicate pigs/treatment were used in the analysis. ZO1 = Zonular occludin-1; MUC2 = Mucin-2; CLDN-4 = Claudin-4; IL8 = Interleukin-8; Casp3 = Caspase-3

Gene expression is presented in Fig. 5 as relative expression [arbitrary units] for ileal and colonic tissue samples. In the ileum, there was a tendency for greater expression of MUC2 (*P* = 0.06), and IL8 (*P* = 0.10) with the HF diet (Fig. 5a). There was no significant effect of Thr or interaction of Thr and fiber on the ileal expression of selected genes. There were no significant treatment effects on the expression of marker genes in the colon (*P* > 0.05; Fig. 5b).

## Discussion

There is a growing research interest in relation to the effects of DF on intestinal health and barrier function. Previous work has demonstrated both direct and indirect roles of DF on intestinal health and barrier function, largely associated with the modulatory effect of DF on microbial environment and the subsequent production of VFA which affects intestinal health and barrier function [6, 30, 31]. Similarly, immune challenge has been shown to affect intestinal health and barrier function as demonstrated by reduced villi height and crypt depth and increased mast cells in LPS challenged weaned pigs [9]. In addition, enteric *E. coli* challenge has been shown to disrupt tight junction assembly and reduce intestinal barrier function in rats [32]. On the other hand, AA are known to play physiological roles as regulators of important functions, apart from participating in protein synthesis [33]. For example, Thr is reported to play an essential role in maintaining mucosal integrity and barrier function by supporting mucin secretion [27, 34]. As such, dietary Thr content has been reported to affect mucin dynamics [18, 35, 36] and support intestinal barrier function and gut health [37]. The aim of the present study was, therefore, to evaluate the independent and combined effects of dietary fiber and Thr on intestinal health and barrier function, using different conditions of immune challenge, including a systemic immune challenge using intravenously administered *E. coli* LPS or enteric challenge with oral *Salmonella* Typhimurium. In the first experiment, we used the L:M absorption test to measure barrier permeability [38–40], as the design allowed for total urine collection which has been reported to improve the accuracy of this method [39–42]. In experiment 2, tissue analysis for histology and gene expression were used to assess barrier function. In both experiments, fecal samples were analyzed for fecal mucin concentration and further estimation of total fecal mucin output based on previously determined dry matter digestibility and fecal output [22].

In experiment 1, we evaluated the effect of systemic LPS injection as an immune stimulatory agent on barrier function and further characterized the role of fiber and Thr on the intestinal barrier function with or without LPS induced immune challenge. Previous work has validated the use of lactulose and mannitol as a credible method of determining intestinal barrier permeability in pigs [39–42]. Movement of lactulose across the intestinal epithelium occurs only through paracellular routes whereas movement of mannitol occurs through both paracellular and transcellular pathways [42]. Therefore, high lactulose recovery is an indicator of increased intestinal permeability [39]. Dietary factors (e.g. fiber) could influence the absorption pathways and metabolism of both these sugars. It is assumed that both sugars will be affected equally by pre-absorptive dietary influences, however, mannitol is regarded more as a normalizing control [42] and, therefore, reporting the L:M ratio is more indicative of response to dietary treatments. In the present study, ISS increased the L:M ratio in LF but not HF-fed pigs which is an indication of reduced barrier function during LPS induced ISS. Previously, the effect of LPS on increased intestinal permeability was reported to occur largely as a result of secondary effect mechanisms, such as oxidative stress to the intestinal epithelium [43, 44]. Observations from the current study suggest that, under conditions of immune stress, intestinal barrier function is disrupted, but feeding a high DF may provide a protective effect on the gut and contribute to improved barrier function. Since increased DF has been reported to increase mucin secretion and potentially contribute to barrier function, total fecal mucin output was measured in the same group of pigs. The absence of an LPS induced effect on total fecal mucin output is contrary to previous studies where systemic LPS was administered [45, 46]. However, the measurement for mucin secretion (i.e., mucosal scrapings) used in those studies was different from the fecal mucin measurement used in the present study. Hence, the lack of effect of LPS on fecal mucin is likely due to the limited direct effects of a systemic LPS induced immune stimulation on intestinal mucin production. However, it should also be noted that fecal mucin analysis may underestimate small intestinal mucin secretion due to the possibility of microbial fermentation of mucus in the hindgut [47, 48]. Thus, fecal mucin analysis may not have detected a mucin-secretory response to LPS in the case where the LPS mucin response was limited to the small intestine. The HF diet increased the total fecal mucin output in the present study consistent with previous reports [49, 50], including reports demonstrating increased ileal mucosal protein losses (e.g. mucin) [16, 51] with increased DF. The increased mucin secretion in response to high DF in the present study may directly contribute to protection against paracellular lactulose transport but may also indicate other concurrent changes in barrier function affecting transcellular permeability in response to systemic LPS. The lack of effect of systemic LPS on mucin production provides support for the lack of additivity between high DF and ISS on Thr requirements as previously reported [22].

In experiment 2, the effects of dietary fiber and Thr on intestinal health and barrier function were further evaluated in pigs challenged with an enteric pathogen (i.e., *Salmonella* Typhimurium).

Measuring the output of mucin in feces provides information on the net output of mucin secreted, largely into the hindgut [52]. The increase in total fecal mucin output with enteric ST inoculation agrees with a previous study that reported increased mucin secretion following immune stress in rats induced by live pathogenic *E. coli* [45]. Although there is some information indirectly relating the impact of immune stimulation on mucus secretion in pigs [53] and chickens [54], the present study demonstrates direct evidence that enteric *Salmonella* Typhimurium challenge increases mucus secretion. This contrasts the lack of fecal mucin output response in LPS challenged pigs reported here. As outlined in our previous work [24], *Salmonella* Typhimurium challenge induced an immune response in pigs, as indicated by clinical response data (rectal temperature, acute phase proteins, and *Salmonella* shedding), without any mitigating effect of high DF. Unlike in experiment 1, we observed an increase in fecal mucin output with *Salmonella* inoculation as compared with systemic LPS administration suggesting significant direct effects of enteric pathogen on intestinal mucin production, which likely reduced the expected effects of high DF on mucin production. As previously reported [24], supplemental Thr improved growth performance in *Salmonella*-challenged pigs, regardless of DF content, however, this increase was less with high DF. In addition, in the current study we observed a fiber by Thr interaction on fecal mucin output, in which supplemental Thr increased fecal mucin output in HF but not LF-fed pigs. It has been shown that mucin production is conserved and prioritized over other functions requiring Thr (e.g., protein anabolism) in pigs [17] and may be mobilized from endogenous proteins (e.g., muscle) to meet increased Thr demands associated with inflammation [55]. The results of the current study provide further evidence that mucin production is prioritized in *Salmonella*-challenged pigs, however, maximal mucin secretion in response to multiple challenges (e.g., disease challenge combined with high DF) may depend on dietary Thr supply. Finally, ileal morphology and goblet cell numbers were measured to ascertain the impact of dietary fiber and Thr on intestinal barrier function. No effects of fiber or Thr were observed on ileal mucosal morphology following the *Salmonella* Typhimurium challenge. A previous report by Hedemann et al. [56] suggested that feeding pigs high insoluble DF increased villi height and improved gut morphology, however, this effect may have been masked by the concurrent *Salmonella* Typhimurium challenge conditions reported here. Goblet cell numbers have been used as indicators of mucus secretion capacity in pigs [27, 50] where increases in goblet cell number, most importantly in the villus indicate an increased capacity [57]. The increase in goblet cell number with HF diets reported here agrees with reports from previous studies [27, 50]. Different fiber sources (e.g. rye bran, oat bran) in the diet of golden hamsters were reported to increase goblet cell numbers in the intestine [58]. Further, Piel et al. [50] fed carboxymethylcellulose, a highly viscous and non-fermentable fiber to weanling pigs and reported a 30% increase in goblet cell number. The tight junction proteins (ZO-1, CLDN4) have been reported to play significant roles in regulating paracellular permeability and barrier function, as such changes in these proteins may indicate changes in intestinal permeability and barrier function [59–61]. However, no significant effect of fiber or Thr on relative expression of other target genes associated with barrier function was observed in the present study. This may be because the pigs used in this study were all challenged with *Salmonella,* as such that the response of the tight junctions to the treatments may have been masked by the effect of the ST challenge. The products of fermentation have been reported previously to improve intestinal health through mechanisms involving modification of microbial environment and composition [6, 41, 62]. Therefore, we measured VFA concentration in intestinal digesta, and the impact of these metabolites in relation to the expression of genes associated with intestinal health and barrier function. As expected, feeding high DF increased significantly the concentration of total VFAs in the cecum and colon digesta. Previous work in rats demonstrated that VFAs (acetate and butyrate) induce mucus secretion in rat colon [63, 64]. Indeed, we observed a trend towards increased MUC2 expression in the ileum of pigs fed HF, which is consistent with the observed increase in goblet cell numbers. Although it has been suggested that mucin dynamics are affected by dietary Thr supply in chickens [65] we did not observe any significant effect of Thr levels on MUC2 gene expression or the expression of other target genes in the present study.

## Conclusion

In conclusion, high DF appears to improve barrier function, as indicated by reduced L:M ratio, through an increase in mucin production capacity (i.e., goblet cell numbers, MUC2 gene expression) and synthesis (i.e., fecal mucin output). Furthermore, the lack of effect of dietary Thr on mucin production in *Salmonella*-challenged pigs fed low-fibre diets but not high-fibre fed pigs provides further evidence that mucin production in the gut is conserved and prioritized and may become limiting when multiple factors affecting mucin production are present. The current results provide information on the interactions of dietary composition and immune status that allow for development of feed programs that maintain performance while promoting gut health.

## Data Availability

The datasets used and analysed during the current study are available from the corresponding author on reasonable request.

## Abbreviations

AA: Amino acid
BW: Body weight
CFU: Colony forming unit
Casp3: Caspase-3
CLDN4: Claudin-4
DF: Dietary fiber
GAPDH: Glyceraldehyde 3-phosphate dehydrogenase
HF: High fiber
IL8: Interleukin-8
ISS: Immune system stimulation
LPS: Lipopolysaccharide
LF: Low fiber
LM: Lactulose:mannitol
MUC2: Mucin-2
RPL19: Ribosomal protein-L19
SID: Standardized ileal digestible
STD: Standard
SUP: Supplemental
ST: *Salmonella* Typhimurium
Thr: Threonine
TDF: Total dietary fiber
VFA: Volatile fatty acid
ZO1: Zonula occludin-1
